# Feasibility of Wearable Devices for Motivating Post-Stroke Patients

**DOI:** 10.3390/s25165204

**Published:** 2025-08-21

**Authors:** Klaudia Marek, Jan Górski, Piotr Karolczyk, Justyna Redlicka, Igor Zubrycki, Elżbieta Miller

**Affiliations:** 1 Department of Neurological Rehabilitation, Medical University of Lodz, Milionowa 14, 93-113 Lodz, Poland; jan.gorski1@student.umed.lodz.pl (J.G.); piotr.karolczyk@student.umed.lodz.pl (P.K.); justyna.redlicka@umed.lodz.pl (J.R.); 2 Institute of Automatic Control, Lodz University of Technology, Stefanowskiego 18, 90-537 Lodz, Poland; igor.zubrycki@p.lodz.pl

**Keywords:** neurorehabilitation, rehabilitation robot, wearable technology

## Abstract

The effectiveness of upper extremity rehabilitation in post-stroke patients significantly depends on patient motivation and adherence to therapeutic regimens. Rehabilitation-assistive technologies, including wearable sensors, have been adopted to facilitate intensive and repetitive exercises aimed at reducing hand dysfunction and enhancing quality of life. Building upon the previously introduced Przypominajka (reminder) system reported in this journal—a wearable sensory glove coupled with a mobile application providing exercise guidance and monitoring—we conducted a feasibility study to evaluate its effectiveness in supporting upper limb rehabilitation. Sixteen post-stroke patients with hemiparesis were equally randomized into experimental and control groups. Both groups performed upper limb exercises for 45 min daily for over two weeks. The experimental group utilized the sensor-equipped glove and tablet-based exercises, whereas the control group followed printed exercise instructions. Clinical improvements were measured using the Fugl–Meyer Assessment–Upper Extremity (FMA-UE), Functional Independence Measure (FIM), and MORE scales. The experimental group demonstrated a minimal clinically important difference (MCID) on the FMA-UE and reported greater overall improvement than the control group. This study confirms the feasibility and potential clinical benefit of supplementing post-stroke rehabilitation with sensor-augmented exercises provided by the previously described Przypominajka device.

## 1. Introduction

Limb motor dysfunction is the most common post-stroke complication, affecting approximately 80% of patients who survive a stroke. Half of the symptoms persist throughout their lives, resulting in disability [1]. Neurological patients require continuous, ongoing rehabilitation and comprehensive care after a stroke. The use of technology supported by sensory and robotic devices can positively impact rehabilitation by providing intensive, specific, and repetitive training to patients and reducing the physical burden on physiotherapists [2]. Up to two-thirds of patients who discontinue rehabilitation after six months experience additional post-stroke deficits and complications [3,4]. Improving hand function after stroke requires a multidisciplinary and multifactorial approach [5]. Studies in primates demonstrate that, to permanently acquire simple but new motor skills, a significant number of exercise repetitions (from 300 to 1500) is required so that changes in motor programming and neuronal reorganization may occur [6]. In the case of brain damage, the flow of information to and from the entire musculoskeletal system is partially or completely impaired, in the case of information both received from and sent to the central nervous system (CNS); therefore, it is possible that it takes an even higher minimum number of repetitions, a longer duration and, above all, a greater frequency of training. For this reason, it is vital for the post-stroke patient to receive individually tailored training that is as intensive as possible [7].

Up to 65% of post-stroke patients experience limb hemiparesis of various levels of severity, as well as other neurological symptoms, depending on the location, size, and extent of the brain injury [8]. In the medical literature, hemiparesis is defined as weakness or the inability to move on one side of the body [8]. It is a severe disorder of motor function characterized by decreased muscle strength [9,10]. Low muscle strength results in a limited range of motion in the joints and impaired balance and coordination of the body, which directly affect the entire rehabilitation process, as the patient is unable to perform many simple activities of daily living and may also experience fatigue more quickly [11]. As a result of hemiparesis, the patient’s posture deteriorates, stretch reflexes are reduced, and voluntary movements are partly or completely impaired [12]. The clinical picture of a patient with hemiparesis is heterogeneous, because, apart from motor disorders, they may also experience sensory disturbances affecting, i.e., touch, hearing, sight, taste, and smell, cognitive and emotional disorders, as well as medical problems related to multimorbidity, especially in the elderly [13]. About 40% of people develop increased muscle tone, or spasticity, one year after a stroke. This condition develops one, three months after a stroke or even later. While muscle strength may gradually improve in hemiparesis, increased muscle tone becomes worse over time. This phenomenon is part of upper motor neuron syndrome [14].

Results from meta-analyses and systematic reviews confirm that rehabilitation supported by technology and robotics can improve the recovery process for both therapists and patients [2,15]. We can observe the special value of using robotics in the case of hand problems. It allows us to perform precise and useful movements in different planes, activating many muscle groups [15]. Robotic therapy can help in the recovery of motor function in patients with subacute conditions. If there are no medical contraindications, rehabilitation should begin in the early stage of stroke, because at this point, due to the phenomenon of brain plasticity, the regenerative potential is high [16]. According to the results of a survey on the use of robotics in rehabilitation by Li et al., specialists emphasize the ease of use of devices, seamless adaptation, and, most importantly, increased therapy and exercise for stroke patients. The main barriers identified by professionals are high costs and human resources [17].

Smartwatches have been successfully applied in the rehabilitation of cardiac patients. These compact devices can monitor heart rhythm safety using electrocardiograms powered by artificial intelligence algorithms [18]. Many patients already utilize smartwatches with exercise apps to support their activity levels [19]. In a study by Fan et al. (2024), smartwatch ownership with health monitoring capabilities led to increased daily step counts and overall physical activity among patients [20].

Analysis of the literature published over the last 15 years reveals a much smaller number of studies on digital technology in neurological patients, including post-stroke patients [21]. In terms of functional abilities, digital technology has been used to record indicators of walking and hand use in post-stroke patients [22]. A critical review of the measurement properties is presented, and factors that hinder and facilitate the implementation of applications in clinical practice and research are identified. Mobile applications are important and dependable for step counting in chronic stroke patients [23].

The growing demand for technology and its rapid advancement, particularly in the development of artificial intelligence, has driven the emergence of mobile health (mHealth). Healthcare demonstrates significant potential for data collection, which can be used to predict health status, guide treatment choices, and support medical research. The use of sensors in healthcare shows great promise in gathering new insights about patients’ conditions [24]. Mobile applications are increasingly being adopted across various branches of medicine, enabling health condition monitoring, fast communication, education, and treatment adherence, especially in the case of chronic diseases [25]. The use of mobile applications by patients is part of digital therapeutics (DTx), a specialized field of healthcare, as defined by the Digital Therapeutics Alliance. The term refers to “the use of information and communication technologies to support health and health-related areas” [26]. Mobile applications in neurology do not have to be about exercise and rehabilitation only. Adcock et al. used mHealth to target secondary prevention of stroke. Adults who have experienced a stroke often use mobile applications on a smartphone to support daily activity [27]. Despite widespread access to digital technologies, there is a paucity of information on the use of mHealth apps in post-stroke patients. The number of platforms available to patients is increasing each year. However, the pace is not as fast as in the case of other diseases [21].

The primary benefits of digital therapies include reducing the costs and time associated with patient visits to the hospital, optimizing communication and administrative tasks within healthcare facilities, and providing continuous monitoring of health indicators for week-to-week comparisons. By constant interaction through an application, patients gain a better understanding of their own limitations and strengths [28]. Mobile applications expand the scope of healthcare services and enhance health management through the use of statistical data, such as physiological measurements [29]. This technology can be employed under the supervision of a specialist, with family support, or independently by the patient [30].

Rehabilitation based on supplementing therapy with sensory devices has great potential for neurological patients with complications involving movement disorders [31]. Previously in this journal, we described the design and technical evaluation of the Przypominajka system—a sensor glove coupled with a tablet application to guide, monitor, and motivate self-training for patients with plegic or paretic hands [32]. That initial work confirmed the system’s on-device machine learning capabilities and positive patient feedback in a particular case study. Building upon that foundation, the primary aim of the current study was to conduct a randomized controlled trial to evaluate the clinical feasibility and therapeutic effectiveness of the Przypominajka system in a cohort of post-stroke patients. Specifically, we sought to determine if this sensor-augmented rehabilitation approach leads to greater improvements in upper extremity function compared to a standard exercise program. The aim of the study was to validate the authors’ device—a prototype wearable sensory device with a tablet application designed for hand exercises for post-stroke patients with hand disorders (paresis/spasticity). We hypothesized that exercises with use of the glove would contribute to changes in self-care, upper limb recovery and functional improvement, and motivation.

## 2. Materials and Methods

### 2.1. Study Design

This study, conducted from February to July 2024, conformed to the standards of the Declaration of Helsinki and received approval from the Bioethics Committee of the Medical University of Lodz (RNN/281/20/KE) for the project “Single-use and personalizable devices for hand rehabilitation” (LIDER/50/0203/L-11/19/NCBR/2020). The study is part of a grant awarded by the National Center for Research and Development of Poland. All the patients included in the study received verbal and written information about the study and gave written informed consent to participate in it. The participants were also informed about the option to withdraw from the study at any time, without consequences. Twenty post-stroke patients were recruited according to the inclusion and exclusion criteria of the study, using 1:1 randomization into an experimental group (EG) and a control group (CG).

In this study, we compared the effects of specially designed exercises with the use of a mobile application and a prototype sensory glove developed for neurological patients with the results achieved when performing the same exercises based on paper instructions and not supported with the device. The patients in the control group were first instructed on how to correctly perform the exercises and then practiced independently. In the experimental group, the study participants received training on how to use the device and how to turn on and operate the application and put on the glove, and then exercised under the supervision of a physiotherapist. Both groups exercised for two weeks, with assessments conducted at three points: before the study began, after the first week, and at the end of the second week.

All the patients enrolled in the study received routine conventional in-ward neurological rehabilitation, five days a week, excluding weekends. The participants were recruited from the Department of Neurological Rehabilitation, Dr. Karol Jonscher Municipal Medical Center, in Łódź, Poland. Only post-ischemic stroke patients with upper limb impairment were included in the study. The study was conducted by physiotherapists in charge of the patients, physicians, and engineers responsible for the equipment and technology. The study was approved by the head of the department.

### 2.2. Participants

**Figure 1:**
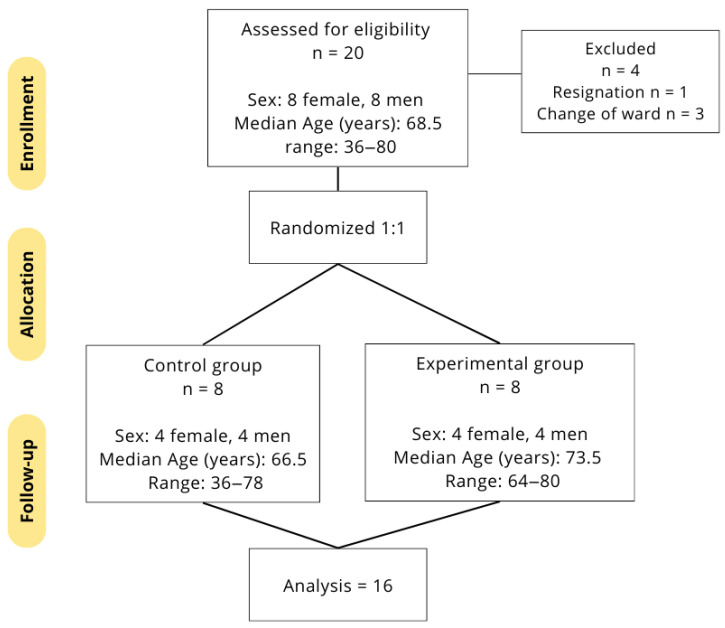
Scheme of the survey structure.

The study included post-ischemic stroke patients with neurological complications (paresis, spasticity) affecting the upper limb. The inclusion criteria for patients in the study were as follows: patients diagnosed with a stroke and MMSE (Mini-Mental State Examination) score above 24—not indicative of significant cognitive impairment, preventing understanding of instructions and participation in research. The exclusion criteria for the study were as follows: inflammatory diseases with fever, severe general condition, systemic diseases, severe cardiovascular conditions (less than 30 days post myocardial infarction, unstable angina pectoris, circulatory insufficiency, severe arrhythmia), impaired ability to understand and perceive, significant general deterioration, impaired understanding of instructions, impaired perception or a significant general decline in cognitive function (with MMSE score as an indicator), uncompensated metabolic and endocrine diseases, and respiratory failure. We recruited 20 patients (ten women and ten men); however, in the course of the study, 4 patients (two women and two men) were excluded—one patient withdrew due to difficulties in understanding the exercises and low motivation, and three patients were transferred to the internal medicine ward due to the development of other stroke-related complications (Figure 1). Before the beginning of the upper extremity training program, the other 16 patients underwent medical assessments.

Sixteen patients (eight women and eight men) participated in the study. Approximately one month after stroke, three patients were in an acute condition, five patients were in the subacute phase for up to six months, and eight patients were in the chronic phase for more than six months. On average, it had been 15.5 months since the patients’ strokes. Paresis occurred with equal frequency (50%) on the left and right sides, corresponding to a 1:1 ratio. The average age of the participants was 67.5 years.

Among the 16 patients participating in the study, as many as 8 were found to have more than one comorbid condition (50%). The comorbid conditions present among participants occurred with the following frequencies: hypertension (62.5%), diabetes (25%), hypercholesterolemia (31.25%), nicotine addiction (50%), hypothyroidism (12.5%), ischemic heart disease (6.25%), sleep apnea (6.25%), and heart attack (6.25%).

### 2.3. Assessment of Patients

Prior to the study, patients were clinically assessed using specialized scales that provided experts with information on their functional state of motivation and the progression/regression of disorders present in the affected upper limb after stroke.

The recruited patients underwent tests using the following clinical scales:

- Fugl–Meyer Assessment–Upper Extremity (FMA-UE);
- Motivation scale (MORE);
- Functional Independence Measure (FIM).

#### 2.3.1. Fugl–Meyer Assessment Upper Extremity (FMA-UE)

Functional assessment of the upper extremity after stroke was assessed using the Fugl–Meyer Upper Extremity Assessment (FMA-UE). This is a commonly used scale that also tests sensorimotor conditions and the associated dysfunction of the upper extremity resulting from stroke [Hassani]. The subscales included in the form allow for accurate and detailed assessment of the affected upper extremity (A—Upper Extremity, B—Wrist, C—Hand, D—Coordination/Speed, H—Sensation, I—Passive Joint Motion, and J—Joint Pain). The FMA-UE has been well known to clinicians for many years as a tool frequently used to measure outcomes. Studies have shown that, based on the FMA-UE scale, it is possible to predict the prognosis of functional status during the first days and weeks after a stroke. Thus, it is possible to plan the rehabilitation process and treatment and evaluate outcomes [33,34,35].

#### 2.3.2. Motivation Scale (MORE)

The MORE scale is a recently published scale invented by Yoshida et al. Their goal was to develop a scale of motivation to rehabilitation for post-stroke patients according to standards based on the Consensus on the Selection of Health Measurement Instruments (COSMIN). The MORE scale addresses two factors that influence patient attitudes—personal factors and those related to social relations [36]. It consists of 17 items that address questions about rehabilitation, capacity, interpersonal relationships, and willingness.

#### 2.3.3. Functional Independence Measure (FIM)

The FIM scale consists of 18 items. It is divided into smaller subscales related to self-care, sphincter control, mobility, locomotion, communication, and social awareness. The score obtained by the patient indicates their level of functional ability [37]. The maximum total score is 126, which corresponds to no problems at the level of functional independence and self-care.

### 2.4. Przypominajka Device and Intervention

The smartwatch-like device was named Przypominajka (Polish for Reminder), as its aim is to constantly remind patients to perform exercises throughout the day. The device consists of a sensory glove that connects to a tablet application via Bluetooth Low Energy. The application displays the exercises the patient should perform, specifically those focused on the wrist joint and forearm. During exercise sessions, the application collects data on how well the exercises are performed. The goal is to achieve five full stars for each exercise, which represents a high-intensity workout with a large number of repetitions. The application randomly selects exercises from a pre-selected set. Each exercise lasts five minutes and is followed by a five-minute break. Each single session includes five exercises. In total, the patient spends 25 min exercising and 20 min resting, as there is no break after the last exercise.

**Figure 2:**
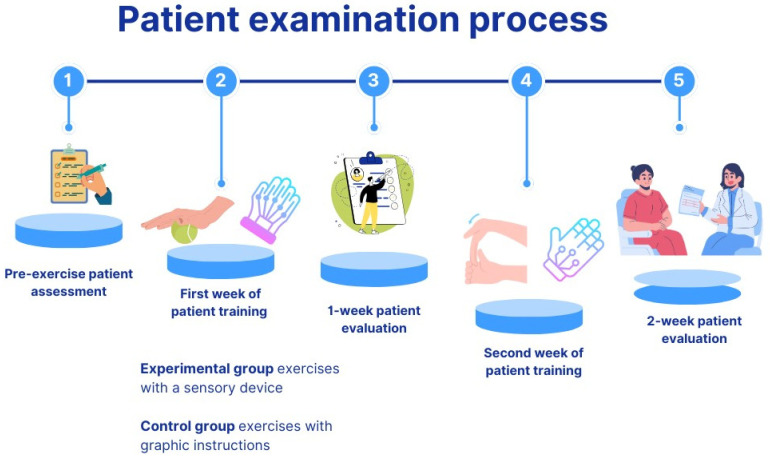
Scheme of the patient-training study.

The study can be divided into five stages. The first stage involves medical assessment of the patients before the beginning of an exercise session. The second stage includes the first week of patient exercise using both graphic instructions and the device. The third stage involves assessment of the patients after the first week of exercise. The fourth stage includes assessment in the second week, and in the final fifth stage, the patients are assessed after completing two weeks of the training sessions (Figure 2).

**Figure 3:**
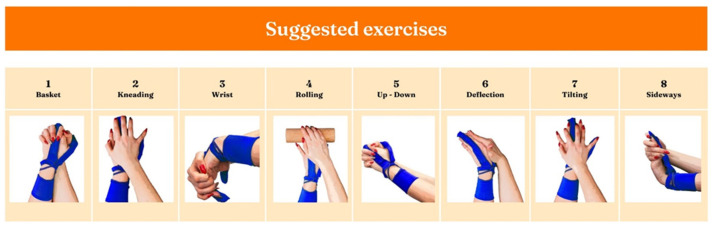
Suggested exercises available in the application.

Przypominajka is a sensory device in the form of a glove worn by a stroke patient on the middle finger, thumb, and wrist of the affected hand. Attached to the device is a tablet with an application that allows the patient to collect points (in the form of stars) that provide information on whether the patient has done enough repetitions and performed the exercises correctly. This is also feedback for the patient, to motivate them to practice further and to signal the correctness of completed tasks. Displayed exercises are randomly selected from among eight suggested movement tasks (Figure 3). When performing an exercise, the patient can receive from zero to five stars, depending on the accuracy of their movements. Five stars correspond to 80% accuracy. The device also allows a margin of error.

**Figure 4:**
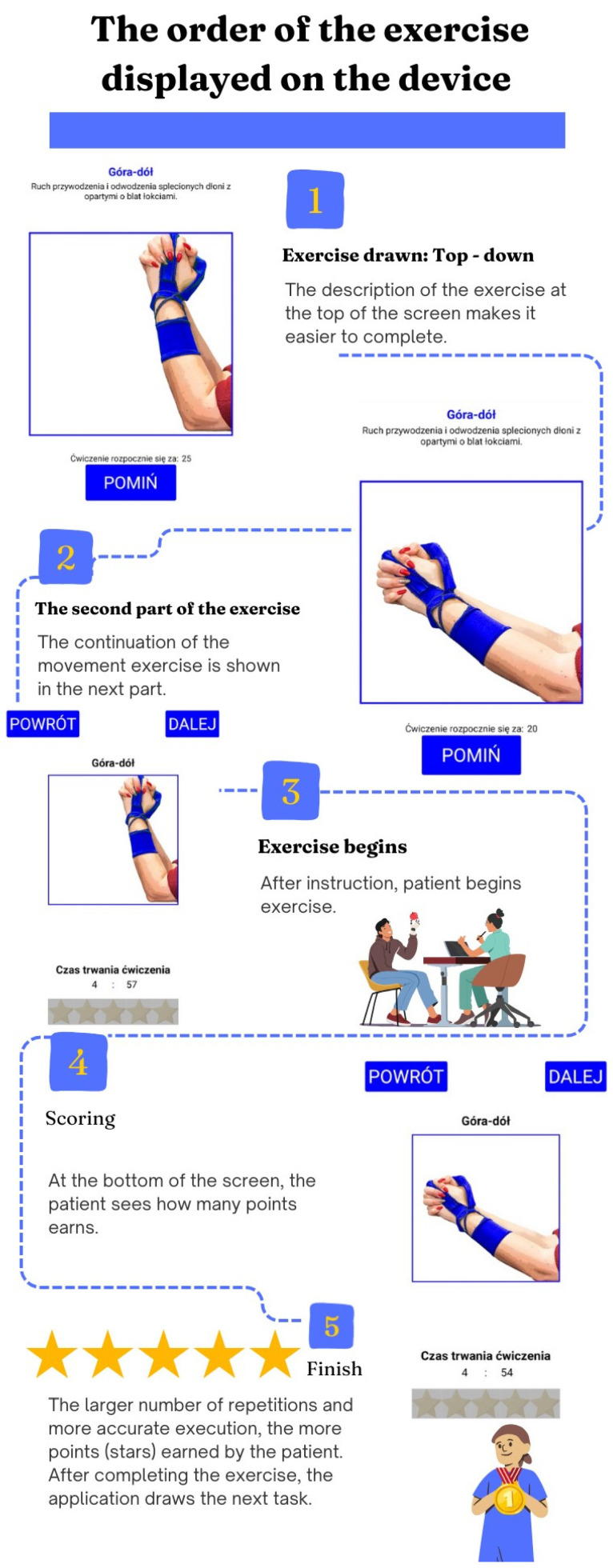
Scheme of application use during the exercise. Góra-dół—Up and down; Ruch przywodzenia i odwodzenia splecionych dłoni z opartymi o blat łokciami—Adduction and abduction movement of interlaced hands with elbows resting on the tabletop; Czas trwania ćwiczenia—Duration of exercise; Powrót—Back; Dalej—Next.

The device is connected to the tablet via Bluetooth Low Energy (BLE). The multimedia application was created using MIT App Inventor 2 (AI2) (Cambridge, MA, USA). The program allows the start and end time of the exercises to be set. The application guides the patient step by step through each session (Figure 4). Once the time is set and the start button is clicked, the patient immediately proceeds to the exercise. The use of such a simple start screen greatly facilitates the use of the device for patients with cognitive function problems, which often occur as a post-stroke complication. After starting the exercise, the application allows the patient a short time to familiarize themself with the next task, which is described with simple commands and supported by a series of pictures illustrating the movement pattern. The tablet beeps at the end of the pause, and the patient clicks the start button to indicate that they are ready to resume the exercise. With this solution, the patient is not forced to control the break time, for fear of omitting the next task.

**Figure 5:**
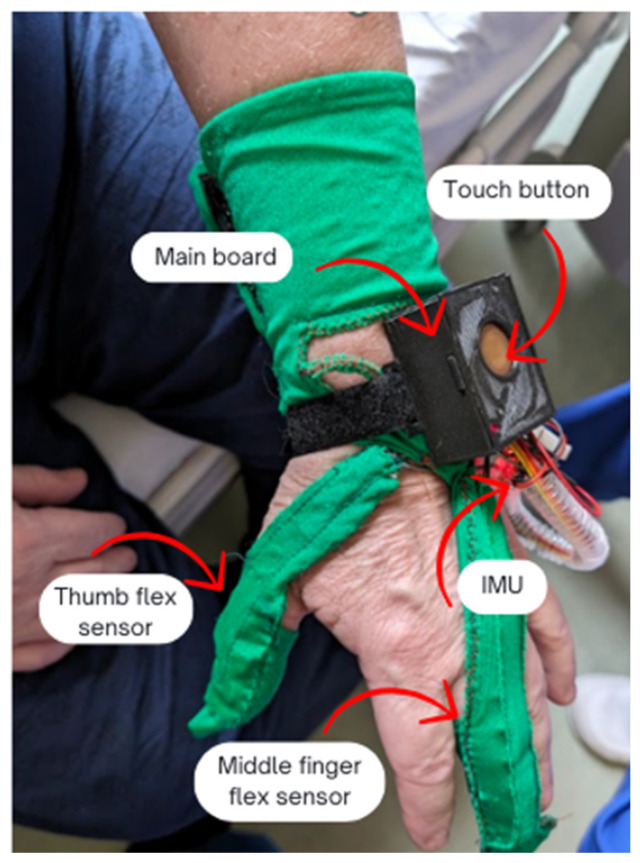
Construction of the device presented on the patient’s hand.

The device is based on a WEMOS LOLIN32 board (WEMOS Electronics, Shenzhen, China) with an ESP32 microcontroller (Espressif Systems, Shanghai, China), (Dual Core Tensilica LX6 240 MHz processor, 520 KB of SRAM, and 4 MB of external flash memory) and a custom-made circuit to collect data from the flexion sensors and from a six-axis IMU (acceleration, rotation) as the patient moves. The flexion sensors are located on the middle finger and thumb (Figure 5). The sensors on the device use a change in the flexion angle to change the resistance of the sensor (flex sensor, Spectra Symbol, Salt Lake City, UT, USA). A step change in the angle causes the change in sensor resistance to disappear. The flexion is measured by a voltage divider-type circuit with a fixed resistor, and the voltage is measured by the ADC circuit of the ESP 32 microcontroller. The sensors are not calibrated; instead, the raw ADC values are applied. The IMU (an MPU6080 integrated accelerometer and gyroscope) displays the angular velocity and acceleration via the I2C interface.

A machine learning-based approach is used to classify a two-second moving window of sensor data, which is sampled at a frequency of 21 Hz. The classification accuracy, which determines if an exercise was performed correctly or incorrectly, was evaluated in a previous study in a set of healthy participants. This evaluation yielded an overall accuracy of 91.3%, with a precision of 90%, a recall of 94%, and an *f*1 score of 91%.

The model, a convolutional neural network (CNN), uses a two-second input vector (containing 42 samples) composed of data from the flex sensors, accelerometer, and gyroscope. Due to the highly nonlinear transformations within the CNN, an ablation study was conducted to evaluate the importance of each sensor’s data. This study revealed that the IMU (MPU6080 accelerometer + gyroscope, InvenSense (TDK) San Jose, CA, USA) data was the most critical feature set. Removing the IMU data caused the model’s accuracy to drop significantly, from 91.3% to 80.3%. Conversely, the flex sensor data was found to be less important, as removing it only caused the categorization accuracy to decrease slightly, to 89.7%. More details can be found in a previous article in which we introduced the device and explained the machine-based approach [32].

## 3. Results

### Statistical Analysis

**Figure 6:**
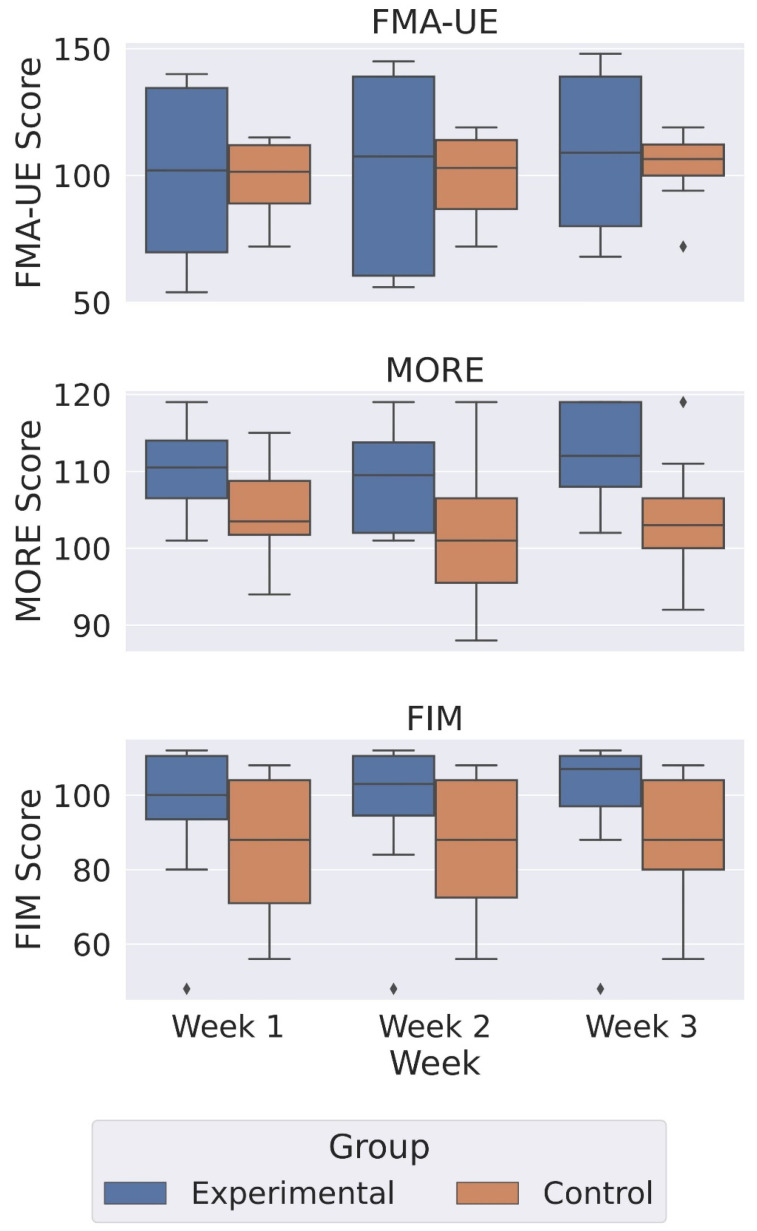
Box plot comparing outcomes for the experimental and control groups at weeks 1/2/3. Boxes denote the interquartile range with median lines; whiskers indicate 1.5 × IQR; individual points represent participants.

Statistical analyses were performed in Python (Statsmodels 0.13.5) for each clinical scale (MORE, FIM, FMA-UE). Within-group changes (week 1 to week 3) were analyzed with paired *t*-tests; between-group differences in change scores were assessed with Student’s *t*-test or Welch’s *t*-test when Levene’s test indicated unequal variances. Normality was evaluated using the Shapiro–Wilk test and visual inspection of Q–Q plots. Equal variance was tested with Levene’s test. Effect sizes were calculated as Cohen’s d for paired or independent samples, with 0.2, 0.5, and 0.8 interpreted as small, moderate, and large, respectively. Statistical significance was defined as *p* < 0.05. The results obtained are presented as boxplots (Figure 6).

Assumption checks indicated that MORE and FMA-UE change scores did not deviate from normality in either group, whereas FIM deviated from normality. Variances were equal for MORE and FIM and unequal for FMA-UE, for which Welch’s *t*-test was applied for between-group comparison (Table 1 and Table 2).

In the case of the MORE scale, the experimental group showed a mean increase of 2.25 points (*p* = 0.164; d = 0.55), while the control group decreased by 1.12 points (*p* = 0.540; d = −0.23). The between-group difference in change scores was not statistically significant (Student’s *t*, *p* = 0.160; d = 0.74). The results for the FIM scale are as follows: the experimental group improved by 3.00 points (*p* = 0.111; d = 0.64), and the control group improved by 1.50 points (*p* = 0.351; d = 0.35). The between-group difference in change scores was not significant (Student’s *t*, *p* = 0.515; d = 0.34). As a sensitivity check for non-normality, non-parametric tests (Wilcoxon within groups; Mann–Whitney between groups) yielded the same conclusions. Nevertheless, the FMA-EU scale is an exception: the experimental group showed statistically and clinically significant improvement (Δ = +9.38 points, *p* = 0.0316; d = 0.95), meeting or closely approaching commonly cited MCID ranges for upper extremity FMA (~9–10 points). The control group improved by 5.38 points (*p* = 0.528; d = 0.23). Levene’s test indicated unequal variances; Welch’s *t* test confirmed that the between-group difference in change scores was not significant (*t*(9.53) = 0.454, *p* = 0.660; d = 0.23).

## 4. Discussion

Our study answers the question of whether a relatively simple device, such as a glove with an attached tablet and an application that randomizes exercises, is useful in post-stroke hand rehabilitation. We find that the wearable Przypominajka device is helpful in the hand rehabilitation process. As an additional therapy, it has been shown to motivate patients and improve hand function and independence. The device allows for high-intensity multifactorial therapy that can improve the motor recovery process in the affected limb [38]. Passive and active movements of the upper limb, suggested as exercises in the application, can enhance motor recovery not only by activating somatosensory stimuli, but also through motor planning and soft tissue properties [16,39].

Smartphones and tablets have been increasingly used by many people, making the implementation of exercise applications relatively easy and inexpensive. Home-based mobile exercise applications for post-stroke patients show great efficacy, which was demonstrated by Chung et al. The results obtained by us confirm the findings of previous studies, namely that a mobile video-based exercise program is more effective than a standard paper-based exercise program. In our study, patients in the experimental group exercised with the application and a sensory glove, which had a significant impact on their sense of motivation in the rehabilitation process. They followed the application’s exercise recommendations more carefully, which resulted in improvements in mobility [40]. An interesting solution was applied by Kim et al., who used scores to motivate patients to achieve better results. The team proposed a self-care rehabilitation system with automatic scoring of the movements made by the patient. Exercises are performed at home and recorded by an application installed on a smartphone. The automated system estimates the result of a completed task after analyzing the uploaded video. The completed task is scored from 0 to 3 points. After the exercise, the patient receives a report on progress or deterioration of the function and exercise recommendations [41]. Our application proposes a scoring system from 0 to 5 points, which allows for a greater variety of possible outcomes. The feedback is provided in real time, based on data fusion from an IMU and flex sensors. Also, by using a glove-based solution, the hand can be accurately tracked even when visual occlusions (due to specificity of exercises) happen.

This study used exercise applications for stroke patients, which may have a positive impact on the phenomenon of digital exclusion among elderly people with neurological conditions [42]. Patients relied on physiotherapists to operate the applications and switch exercises due to their lack of confidence in their ability to use such applications. This attitude, often observed among older adults, stems from digital exclusion, defined as inequality in access to and the ability to use information and communication technologies (ICTs), such as the Internet [43]. Digital disadvantage is most prevalent among elderly people, who make up the largest proportion of this group worldwide [44]. Despite the widespread use of smartphones, there is still a noticeable gap between particular age groups in terms of using ICT services [45]. In Poland, 82% of seniors aged 55–74 years show a low level of digital literacy. According to 2023 data, 75% of Polish seniors aged 60–74 years have never used the Internet [46]. Research has linked digital exclusion to poor physical and mental health in older adults [47,48]. The non-use of digital services is not only due to a lack of skills, but also to health-related barriers that limit older adults’ ability to learn new technologies. These barriers include visual impairments, as well as motor impairments caused by musculoskeletal or neurological conditions, dementia, and difficulties with activities of daily living (ADLs) [49]. Factors contributing to digital exclusion include a lack of Internet skills, an inability to afford Internet access or digital devices, and a refusal to use the Internet, either by the individual or their family [43,50,51]. Older adults who are excluded from digital services are more likely to develop functional dependency, regardless of whether they live in high- or low-income countries [44].

The subacute phase begins approximately seven days after stroke. This phase is characterized by the onset of regenerative processes [52]. The subacute and chronic phases are associated with specific post-stroke repair processes, mainly neoangiogenesis and neurogenesis, respectively [53]. In the subacute phase, brain plasticity and neurorepair aim at restoring the lost core of brain tissue after stroke [54]. The chronic phase begins four weeks after stroke onset [53]. Scientific evidence in humans suggests that there is a spontaneous recovery of function within the first three months, which results from a complex pattern of brain reorganization [55]. In our study, we recruited patients in the subacute and chronic post-stroke phases and presented the rationale for prolonging rehabilitation. A greater improvement on the clinical scales was observed in the patients exercising with a sensory glove, while the control group, using graphic exercise instructions, showed a smaller increase on the FIM scales and in the FMA-UE total score.

Even in 2024, there is still a lack of information on the most effective neurological rehabilitation for post-stroke patients that could maximize the recovery process. We know that rehabilitation in the subacute phase can provide great functional benefits and reverse motor complications [56,57]. Rehabilitation and exercise should not be abandoned in the chronic phase, even though we know that the most significant recovery of motor function occurs within the first six months after stroke. The rate of recovery and return of function slows down significantly after six months; however, some improvement is still possible [58]. Rehabilitation using technology, including a smartwatch or robot, in conjunction with conventional rehabilitation can yield satisfactory outcomes in both the subacute and chronic phases [59,60]. This type of rehabilitation is easy to perform, can be delivered in non-hospital settings, and is widely available to a wide range of patients. It affects motor learning ability and improves hand function [61,62]. Many studies present robots, including exoskeletons that are large, heavy, and driven by rigid joints, that perform movement during training tasks [63]. This gives the option of performing precise movements; however, for many patients it is also a limitation, and there is often interference in the interaction [64]. Our device is made of a soft material, so its weight, even with cables attached, is low. It is handy, cheap, and easy to manufacture. It can be adjusted to fit the size of the patient’s hand and can be used in different environments, such as in hospitals or at home. The COVID-19 pandemic [65] has shown us the importance of creating opportunities for full or continued rehabilitation at home. In this field, there is a need for strategies and technological support to ensure continuity of therapy for patients. A study by Chae et al. (2020) conducted during the pandemic showed that a home care system using a commercially available smartwatch can facilitate participation in home exercise and improve hand functional outcome in post-stroke patients [65]. The application for therapy that we propose is easy to use and intuitive, as subsequent steps are guided by arrows. Przypominajka is available for all devices based on the Android operating system. The application does not have unnecessary options or functions that could be confusing to the patient and cause problems with returning to the main panel and exercises.

The patients in the experimental group were given tablets with a mobile exercise application during the trial. All of the subjects had previous exposure to digital technology, as they had personal smartphones with mobile Internet and used them daily. One patient who withdrew from the study at his own request had no active contact with technology. He did not own a smartphone and felt confused when using a mobile application on a tablet. A similar form of glove was proposed by Nathan et al. to support grip function in self-care therapy for post-stroke patients. The glove was used to measure thumb and finger joint angles and grip. The device assisted the patient in performing a grasping movement. The study demonstrated the potential of measuring grip during functional tasks performed by patients. However, a significant number of researchers designing gloves use thick materials that cover the entire hand, making it difficult for the patient to perform a movement [66]. Wearable technology has proven to be a promising adjunctive therapy that can be delivered at home. Maintenance costs are low, and high availability allows for individualized rehabilitation [67].

Stroke patients often become angry and frustrated when it becomes clear that they have not achieved all their goals and have not regained their physical form. Rehabilitation should be designed in a way that motivates the patient and has a visible effect on their condition. If the patient is not motivated to exercise, even with the support of the best physiotherapists, they will not be able to achieve a satisfactory rehabilitation outcome [68]. Rehabilitation of stroke patients is long-term; it often takes time to obtain satisfactory results. Therefore, it is believed that rehabilitation of post-stroke patients is a lifelong process [69]. Currently, there is an ongoing discussion in the research community on how to increase motivation for rehabilitation in post-stroke patients [70]. In our study, we assessed motivation using the MORE scale. At the beginning of the therapy process, the patients in both groups showed low motivation to exercise, which increased in the experimental group during the second week of training compared to the control group; however, the level of motivation was still not very high. The results presented here confirm previous assumptions by other authors that motivating post-stroke patients to participate in rehabilitation can sometimes be difficult, so a social and psychological intervention model should be introduced in the future to provide social and psychological support to patients [68].

Our study has some limitations. It was carried out in 16 patients after a stroke, and the participants exercised for two weeks (14 days) only. More reliable results and conclusions can be drawn with a longer study period and a larger sample size. Due to the small sample size and short time period, it may not be possible to detect an existing effect, resulting in low statistical power. The potential for overestimating effects increases with a small number of patients, so large, randomized, open-label studies and blinded trials should be conducted after small pilot studies. The FMA-EU scale is an exception, as the experimental group improved by 9.38 points (*p* = 0.0316; d = 0.95, large). This exceeds commonly cited minimal detectable change (MDC) values for chronic stroke (~5.2 points) and meets or closely approaches minimal clinically important difference (MCID) estimates for subacute stroke (≈9–10 points), supporting both clinical and statistical significance [56,71]. The control group improved by 5.38 points (*p* = 0.528; d = 0.23), below MCID thresholds. Levene’s test indicated unequal variances (*p* = 0.0058); Welch’s *t*-test confirmed that the between-group difference was not significant (*t*(9.53) = 0.454, *p* = 0.660; d = 0.23). Regarding patient outcomes on the FIM scale, the experimental group improved by 3.0 points (*p* = 0.111; d = 0.64, moderate) and the control group by 1.5 points (*p* = 0.351; d = 0.35). Both changes are well below reported MCID values for stroke rehabilitation (≈22 points for total FIM; ≈17 points for the Motor subscore) [72], indicating that the observed gains are unlikely to be clinically meaningful over a two-week interval. Changes in FIM scores violated the normality assumption in both groups but had equal variances; non-parametric Wilcoxon and Mann–Whitney tests yielded the same conclusions as the parametric tests. In the case of the MORE scale, the experimental group increased by 2.25 points (*p* = 0.164; d = 0.55, moderate), while the control group decreased by 1.12 points (*p* = 0.540; d = −0.23). No between-group difference was statistically significant (*p* = 0.160; d = 0.74). To our knowledge, no MCID for the MORE scale has been published, so clinical significance must be inferred from effect size and direction of change rather than a threshold [73]. There are several reasons why *p*-values differ across scales. The FMA-UE measures impairment-level motor recovery and is typically more sensitive to short-duration, targeted upper-limb training, which explains the statistically and clinically significant within-group improvement in the experimental group. In contrast, the FIM reflects broader functional independence and requires larger or longer-term gains to surpass MCID thresholds, consistent with the small, non-significant changes observed here. Motivation (MORE) improved directionally with a moderate effect size, but large variability in score changes, particularly in the control group for FMA-UE, and the small sample size reduced the statistical power for between-group differences. These patterns are consistent with the differing constructs, responsiveness, and MCID benchmarks of the three scales.

Another limitation that arose during the study was the number of exercises presented by the application. According to the patients, it was low, and frequent repetition of the same exercises that had already appeared earlier in the session may have led to patient boredom. It was not uncommon for patients to ask for the exercise to be selected again. The patients also commented on the lack of stretching exercises, which are useful for spastic paresis. The digital exclusion of older patients seemed to be another limitation. Despite the large and bold font in the application, the patients were reluctant to use the tablet on their own. Before starting the exercises, they were given detailed instructions on how to use the application, and they read all the messages in the application on their own. Unfortunately, this was not enough to encourage older patients to use the application without assistance. They did not feel confident enough and sometimes had difficulty reading the words in the application. This is not surprising, as stroke patients often show cognitive impairment at various visuospatial levels, problems with orientation, attention focus, or memory. Delayed recovery of the above functions is an inherent part of the recovery process after stroke. It is related to cerebral ischemia and hypoxia during the development of a stroke lesion [74,75]. The prototype glove proved difficult for most patients to wear. A physiotherapist was always available during training; however, staff encouraged the patients to use the device themselves, with the aim of achieving independence. Unfortunately, in several cases, patients required assistance, cables got tangled, and the fitting process was prolonged. In one case, a cable broke, and the device had to be checked and repaired quickly. It should be noted that our device is a prototype with components printed on a 3D printer. These findings confirm and expand upon the ergonomic challenges identified in our initial case study. The difficulty patients experienced in putting on the glove and the prototype’s durability issues highlight that, while the sensing and software concepts are viable, significant hardware refinement is necessary for independent use. Furthermore, a key technical limitation noted in our previous work was a drop in the classification accuracy of the machine learning model when applied to new users not included in the original training set. The positive clinical outcomes observed in this trial were achieved despite this known imperfection in the scoring algorithm’s generalizability. This suggests that even a directionally correct, encouraging feedback system can be motivating and effective, but it also underscores the potential for even greater gains if the system incorporated methods for rapid personalization or on-device learning for new patients.

Our study has shown that additional training with a sensory glove and application leads to better scores on clinical scales. This means improvements in hand function, self-care, and functional independence and a greater motivation to exercise. The observed improvement was not significant in all cases, which clearly indicates that the challenge faced by physiotherapists and occupational therapists in relation to rehabilitation of the wrist in post-stroke patients is difficult. In terms of degrees of freedom, after the shoulder, the wrist is the second most complex upper limb joint. The complexity of its structure also makes it difficult to rehabilitate [76]. The use of adjunctive therapy with a wearable device in the form of a sensory glove with an application has added variety to routine rehabilitation and motivated patients to exercise. However, the therapy needs to be refined to be more useful in rehabilitation.

After completing the study, all patients received graphic instructions for the exercises they performed during the pilot program. It is planned to increase the number of participants in the future and conduct the study simultaneously in several international centers, which will allow for more reliable conclusions to be drawn regarding the future use of the device for stroke patients with hand paresis.

## 5. Conclusions

Additional upper limb exercises using an exercise application and a sensory glove that controls the precision and quantity of the exercises can have a positive impact on the post-stroke rehabilitation process as an adjunct therapy. Increasing the number of exercises for neurological patients led to better results on clinical scales, which corresponds to improved hand function, independence, and functional autonomy, or increased motivation for rehabilitation. Different solutions should be sought to reduce digital exclusion and encourage older people to diversify their neurological rehabilitation.

## Figures and Tables

**Table 1:** Assumption checks and between-group comparisons.

Scale	Shapiro–Wilk *p* (Exp) (a)	Shapiro–Wilk *p* (Ctrl) (a)	Levene *p* (b)	Student’s *t*-Test *p* (c)	Welch’s *t*-Test *p* (d)	Cohen’s d (e)
MORE	0.437	0.397	0.519	0.160	—	0.74
FIM	0.004	<0.001	0.436	0.515	—	0.34
FMA-UE	0.821	0.177	0.0058	0.657	0.660	0.23

(a) Shapiro–Wilk test for normality of change scores within each group; *p* > 0.05 indicates no evidence against normality. (b) Levene’s test for equality of variances between groups; *p* > 0.05 indicates equal variances. (c) Between-group comparison of change scores assuming equal variances. (d) Between-group comparison of change scores, not assuming equal variances (reported when Levene’s *p* < 0.05). (e) Cohen’s d effect size for the between-group difference in change scores (small ≈ 0.2, moderate ≈ 0.5, large ≥ 0.8).

**Table 2:** Within-group paired *t*-tests.

Scale	Group	*n*	Mean Δ	SD Δ	*t* (*n* − 1)	*p*-Value	Cohen’s d (f)
MORE	Experimental	8	2.25	4.10	−1.55	0.164	0.55
MORE	Control	8	−1.12	4.94	0.64	0.540	−0.23
FIM	Experimental	8	3.00	4.66	−1.82	0.111	0.64
FIM	Control	8	1.50	4.24	−1.00	0.351	0.35
FMA-UE	Experimental	8	9.38	9.90	−2.68	0.0316	0.95
FMA-UE	Control	8	5.38	22.88	−0.66	0.528	0.23

(f) Cohen’s d for paired samples calculated as mean change divided by the standard deviation of change scores.

## Data Availability

The article contains all of the data.
